# A three-dimensional finite element model of cAMP signals

**DOI:** 10.1016/j.finmec.2021.100041

**Published:** 2021-09-04

**Authors:** R. Warren, T.C. Rich, S.J. Leavesley, A.-V. Phan

**Affiliations:** a Department of Mechanical, Aerospace and Biomedical Engineering, University of South Alabama, Mobile, AL 36688, USA; b Center for Lung Biology & Department of Pharmacology, University of South Alabama, Mobile, AL 36688, USA; c Department of Chemical and Biomolecular Engineering, University of South Alabama, Mobile, AL 36688, USA

**Keywords:** Second messenger signals, cAMP intracellular signaling, Endothelial cells, Pulmonary vasculature, Finite element analysis

## Abstract

This paper presents a three-dimensional finite element model for cyclic adenosine monophosphate (cAMP) signaling. Governing equations for the synthesis, diffusion, and degradation of cAMP were numerically implemented using the finite element method. Simulated results were displayed as time course plots of cAMP concentrations at selected nodes within the discretized geometry. The validity of the finite element model was assessed by comparing simulated results against analytical or other numerical solutions of cAMP concentration distribution for a spherical cellular volume. An endothelial cell was also simulated using its discretized geometry obtained from microscopic cellular cross-sectional images. Simulated solutions using the spherical cellular volume produced near identical cAMP concentration plots to the analytical solutions and were in good agreements with numerical results obtained from VCell, an existing software package for modeling cell biological systems. The validated 3-D finite element model was then employed to simulate the cAMP signaling pathway within a pulmonary microvascular endothelial cell geometry.

## 1. Introduction

Although enzymatic control of the cyclic adenosine monophosphate (cAMP) signaling pathway is well understood, the way in which information is encoded within cAMP signals is not as well defined. cAMP is a second messenger in intracellular signaling involved in the regulation of numerous cellular functions. These functions include cellular proliferation, differentiation, and gene expression [1,2]. Synthesis of cAMP occurs typically at the plasma membrane in response to stimulated adenylyl cyclase (AC) activity. Degradation of cAMP is due to phosphodiesterase (PDE) enzyme activity. There is evidence of correlations between cAMP levels and disease. For example, in vitro infections with HIV have shown T cells with higher concentrations of cAMP [3]. Pharmaceuticals regulating cAMP levels have been developed to treat diseases such as diabetes, hypertension, and asthma [3–6]. Phosphodiesterase inhibitors, in particular, have been proven as successful treatments for central nervous system damage [7].

With only rudimentary knowledge of information encoding within intracellular signals, we have limited understanding of the subcellular localization, kinetics, and frequency of cAMP signals [8,9]. Numerical models for predicting spatial distributions of cAMP and other second messengers have been developed. A fourth order Runge-Kutta MATLAB script was used to simulate cAMP distributions near the plasma membrane of HEK-293 cells [10–12]. A large-scale stochastic model was also developed to model the subcellular localization of cAMP signals in HEK-293 cells using the software NeuroRD [13]. Both the models and study outcomes have been summarized [14]. The program Virtual Cell [15], or VCell, is an intracellular modeling software developed by the University of Connecticut. It uses the Finite Volume Method to simulate deterministic partial differential equation systems onto uploaded cellular geometries [15].

This work focuses on the implementation of the finite element method (FEM) for three-dimensional modeling of the cAMP signaling pathway. The finite element method is a numerical engineering technique useful in complex geometries. It relies on the discretization of the geometry and the conversion of the partial differential equations governing the system into a system of algebraic equations. A two-dimensional finite element model had been developed and reported in our previous work [16] where more information about related works on cAMP intracellular signaling can be found.

The three-dimensional finite element model used equations governing the synthesis, diffusion, and degradation of cAMP. Four node tetrahedral elements were used to discretize the cellular geometries. Simulations were then run with initial conditions and parameters to model the spatial and temporal dynamics of the cAMP signaling pathway. Both spherical and realistic cellular geometries have been modeled. Cellular geometries were obtained from endothelial cross-sectional cell image sequences that were converted into a solid, finite element meshed volume. The finite element model ran off MATLAB scripts.

The 3-D finite element simulations were validated through comparison of available analytical solutions and of resulting simulations previously done using the software VCell [17]. The validated 3-D FEM model was then utilized for simulating cAMP intracellular signaling within a pulmonary microvascular endothelial cell (PMVEC).

In a previous work from our group, a 3-D FEM model was developed [18]. However, that model was based upon a linear approximation of the Michaelis-Menten enzyme kinetics about the initial value of cAMP concentration. As a result, the model in [18] is only accurate for low cAMP concentrations. On the other hand, the 3-D FEM model proposed in this work utilized a quasi-linearization of the Michaelis-Menten kinetics. This technique is based on a linear approximation about the cAMP concentration level at any given time. Despite the fact that the quasi-linearization requires some iterations to converge, the technique produces an accurate estimate of PDE reactions at any levels of cAMP concentration. In addition, the 3-D model developed in this work is able to simulate a variety of AC and PDE activities anywhere within the cell, as well as the instants when these activities start.

## 2. Governing equation

For 3-D cAMP intracellular signaling, its governing equation describing cAMP synthesis, diffusion and degradation is generally given as (see [19,20])

(1) $$\frac{\partial C}{\partial t}=\{\begin{array}{ll} D\nabla^{2}C & \text{if}\;t<t_{s}\\ D\nabla^{2}C+E_{AC}(x,y,z) & \text{if}\;t_{s}\leq t<t_{d}\\ D\nabla^{2}C+E_{AC}(x,y,z)-M(C) & \text{if}\;t\geq t_{d} \end{array}$$

where *C* = *C*(*t, x, y, z*) is the cAMP concentration at time *t* and location (*x,y,z*), *D* is the diffusion coefficient, *E*_*AC*_ is the cAMP synthesis function, *M*(*C*) is the Michaelis-Menten cAMP degradation function, and *t*_*s*_ and *t_d_* are the time points when AC activity (cAMP synthesis) and PDE activity (cAMP degradation) start taking place, respectively. In this work, PDE activity is assumed to occur after or at the same time of AC activity $\left(t_{s}\leq t_{d}\right)$.

Under the steady-state assumption,

(2) $$M(C)=\frac{V_{\max}C}{K_{M}+C}$$

where *V*_max_ is the maximum cAMP hydrolysis rate, and *K*_*M*_ is the Michaelis-Menten constant for cAMP binding to PDE.

As *M*(*C*) is a nonlinear function in *C*, an iterative method will need to be employed in the FEA of the model given by Eq. (1). The iterative technique adopted in this work is based on a quasi-linearization of *M*(*C*) about *C* = *C*_1_:

(3) $$M(C)=M\left(C_{1}\right)+{\frac{\text{d}M}{\text{d}C}|}_{C=C_{1}}\left(C-C_{1}\right)\;\;=\frac{V_{\max}K_{M}}{{\left(K_{M}+C_{1}\right)}^{2}}C+\frac{V_{\max}C_{1}^{2}}{{\left(K_{M}+C_{1}\right)}^{2}}$$

To accurately evaluate Eq. (2) using its quasi-linearization (3), at a given time and location, the iteration must be carried out until the difference between the predicted *C*_1_ and the solution *C* resulting from solving the third equation of system (1) satisfies a chosen convergence criterion.

The possible boundary conditions are

- *C* is specified;
- Normal derivative (concentration flux) is prescribed along a boundary surface,
(4) $$D\frac{\partial C}{\partial n}=\beta$$
  where **n** is the normal vector to the boundary surface and *β* is a constant. For the proposed FEA model, this concentration flux *β* can be used to simulate AC activities in the plasmalemmal or perinuclear region of a cell.

## 3. Finite element implementation of the governing equation

To use the Galerkin approximation, we first discretize the cellular geometry into a number of elements. For each element, we multiply Eq. (1) by the shape functions *N_i_* (*i* = 1, 2, …, *n*, where *n* is the number of nodes of the chosen type of element) selected as weighting functions and then integrate it over the volume *V* of the element as follows:

(5) $$\begin{array}{ll} \int_{V}\left(\frac{\partial C}{\partial t}-D\nabla^{2}C\right)N_{i}\text{d}V=0 & \text{if}\;t<t_{s}\\ \int_{V}\left(\frac{\partial C}{\partial t}-D\nabla^{2}C-E_{AC}(x,y,z)\right)N_{i}\text{d}V=0 & \text{if}\;t_{s}\leq t<t_{d}\\ \int_{V}\left(\frac{\partial C}{\partial t}-D\nabla^{2}C+aC-b\right)N_{i}\text{d}V=0 & \text{if}\;t\geq t_{d} \end{array}\}$$

where $\nabla^{2}$ is the Laplacian, and

(6) $$a=\frac{V_{\max}K_{M}}{{\left(K_{M}+C_{1}\right)}^{2}}\;b=E_{AC}(x,y,z)-\frac{V_{\max}C_{1}^{2}}{{\left(K_{M}+C_{1}\right)}^{2}}$$

By using Gauss’s divergence theorem on the diffusion term and the boundary conditions (see, *e.g.*, [21] for more details), one obtains the following weak form in 3-D:

(7) $$\begin{array}{ll} \int_{V}\left(\frac{\partial C}{\partial t}N_{i}+D\left(\frac{\partial C}{\partial x}\frac{\partial N_{i}}{\partial x}+\frac{\partial C}{\partial y}\frac{\partial N_{i}}{\partial y}+\frac{\partial C}{\partial z}\frac{\partial N_{i}}{\partial z}\right)\right)\text{d}V=0 & \text{if}\;t<t_{s}\\ \int_{V}\left(\frac{\partial C}{\partial t}N_{i}+D\left(\frac{\partial C}{\partial x}\frac{\partial N_{i}}{\partial x}+\frac{\partial C}{\partial y}\frac{\partial N_{i}}{\partial y}+\frac{\partial C}{\partial z}\frac{\partial N_{i}}{\partial z}\right)\right)\text{d}V=\beta \int_{S_{n}}N_{i}\text{d}S+\int_{V}E_{AC}N_{i}\text{d}V & \text{if}\;t_{s}\leq t<t_{d}\\ \int_{V}\left(\frac{\partial C}{\partial t}N_{i}+D\left(\frac{\partial C}{\partial x}\frac{\partial N_{i}}{\partial x}+\frac{\partial C}{\partial y}\frac{\partial N_{i}}{\partial y}+\frac{\partial C}{\partial z}\frac{\partial N_{i}}{\partial z}\right)+aCN_{i}\right)\text{d}V=\beta \int_{S_{n}}N_{i}\text{d}S+\int_{V}bN_{i}\text{d}V & \text{if}\;t\geq t_{d} \end{array}\}$$

where *S*_*n*_ is the face of the element over which its concentration flux is specified.

In this equation, the concentration *C* of cAMP is interpolated over the element from the nodal values $c_{1},c_{2},\ldots ,c_{n}$ using the shape functions *N*_1_, *N*_2_, …, *N*_*n*_ as follows:

(8) $$C=\left[\begin{array}{c} N_{1}\;N_{2}\cdots N_{n} \end{array}\right]\left\{\begin{array}{c} c_{1}\\ c_{2}\\ \vdots \\ c_{n} \end{array}\right\}=[N]\{c\}$$

Thus the time derivative of *C* and its gradients are given by

(9) $$\frac{\partial C}{\partial t}=\left[\begin{array}{c} N_{1}\;N_{2}\cdots N_{n} \end{array}\right]\left\{\begin{array}{c} {\dot{c}}_{1}\\ {\dot{c}}_{2}\\ \vdots \\ {\dot{c}}_{n} \end{array}\right\}=[N]\{\dot{c}\}$$

(10) $$\left\{\begin{array}{c} \frac{\partial C}{\partial x}\\ \frac{\partial C}{\partial y}\\ \frac{\partial C}{\partial z} \end{array}\right\}=\left[\begin{array}{cccc} \frac{\partial N_{1}}{\partial x} & \frac{\partial N_{2}}{\partial x} & \cdots & \frac{\partial N_{n}}{\partial x}\\ \frac{\partial N_{1}}{\partial y} & \frac{\partial N_{2}}{\partial y} & \cdots & \frac{\partial N_{n}}{\partial y}\\ \frac{\partial N_{1}}{\partial z} & \frac{\partial N_{2}}{\partial z} & \cdots & \frac{\partial N_{n}}{\partial z} \end{array}\right]\left\{\begin{array}{c} c_{1}\\ c_{2}\\ \vdots \\ c_{n} \end{array}\right\}=[B]\{c\}$$

Substitution of Eqs. (8)–(10) into Eq. (7) results in,

(11) $$\begin{array}{ll} \left[K_{1}\right]\{\dot{c}\}+\left[K_{2}\right]\{c\}=\{0\} & \text{if}\;t<t_{s}\\ \left[K_{1}\right]\{\dot{c}\}+\left[K_{2}\right]\{c\}=\left\{R_{1}\right\}+\left\{R_{2s}\right\} & \text{if}\;t_{s}\leq t<t_{d}\\ \left[K_{1}\right]\{\dot{c}\}+\left[\left[K_{2}\right]+\left[K_{3}\right]\right]\{c\}=\left\{R_{1}\right\}+\left\{R_{2b}\right\} & \text{if}\;t\geq t_{d} \end{array}\}$$

where

(12) $$\left[K_{1}\right]=\int_{V}{\left[N\right]}^{\text{T}}[N]\text{d}V$$

(13) $$\left[K_{2}\right]=\int_{V}{\left[B\right]}^{\text{T}}[\kappa ][B]\text{d}V$$

(14) $$[\kappa ]=D\left[\begin{array}{ccc} 1 & 0 & 0\\ 0 & 1 & 0\\ 0 & 0 & 1 \end{array}\right]$$

(15) $$\left[K_{3}\right]=a\int_{V}{\left[N\right]}^{\text{T}}[N]\text{d}V$$

(16) $$\left\{R_{1}\right\}=\beta \int_{S_{n}}{\left[N\right]}^{\text{T}}\text{d}S$$

(17) $$\left\{R_{2s}\right\}=E_{AC}\int_{V}{\left[N\right]}^{\text{T}}\text{d}V\;\text{if}\;t_{s}\leq t<t_{d}$$

(18) $$\left\{R_{2b}\right\}=b\int_{V}{\left[N\right]}^{\text{T}}\text{d}V\;\text{if}\;t\geq t_{d}$$

**Fig. 1.**
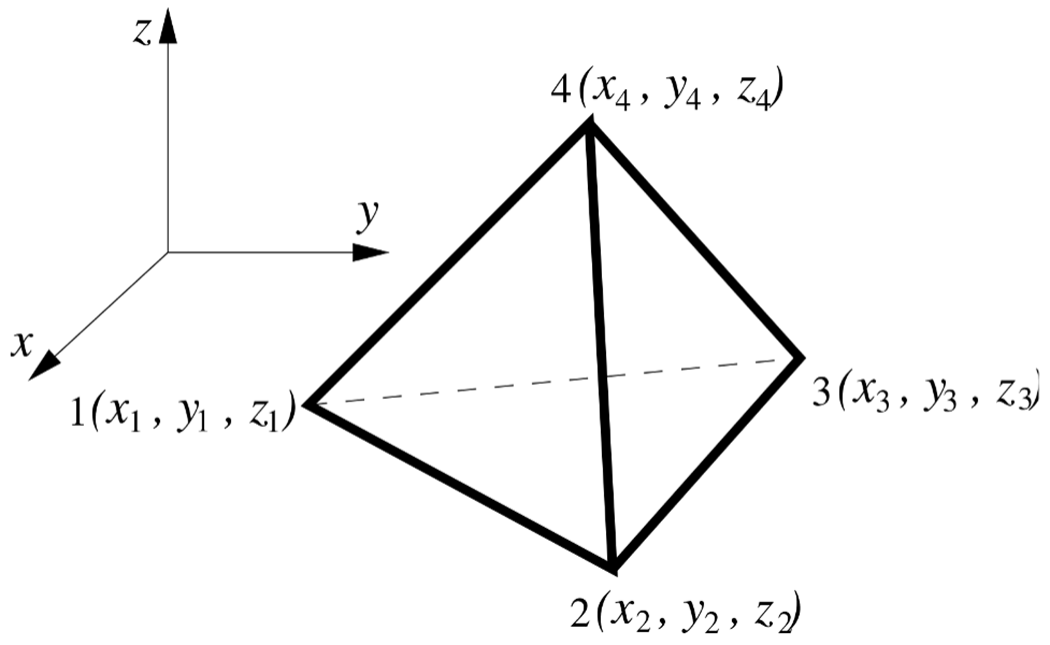
A four-node tetrahedral element.

In this work, the four-node tetrahedral element (see Fig. 1) was selected for a 3-D numerical implementation of Eq. (11). The shape function matrix of this element is known to be

(19) $$[N]=\left[\begin{array}{cccc} 1-\xi -\eta -\zeta & \xi & \eta & \zeta \end{array}\right]$$

where *ξ, η* and *ζ* are the coordinates of the natural coordinate system.

By using these shape functions in Eqs. (12), (13), (15)–(18), one gets

(20) $$\left[K_{1}\right]=\frac{V}{20}\left[\begin{array}{cccc} 2 & 1 & 1 & 1\\ 1 & 2 & 1 & 1\\ 1 & 1 & 2 & 1\\ 1 & 1 & 1 & 2 \end{array}\right]$$

(21) $$\left[K_{2}\right]=V{\left[B\right]}^{T}[\kappa ][B]$$

where

(22) $$\left[B\right]={\left[\begin{array}{ccc} x_{2}-x_{1} & y_{2}-y_{1} & z_{2}-z_{1}\\ x_{3}-x_{1} & y_{3}-y_{1} & z_{3}-z_{1}\\ x_{4}-x_{1} & y_{4}-y_{1} & z_{4}-z_{1} \end{array}\right]}^{-1}\left[\begin{array}{cccc} -1 & 1 & 0 & 0\\ -1 & 0 & 1 & 0\\ -1 & 0 & 0 & 1 \end{array}\right]$$

(23) $$\left[K_{3}\right]=a\left[K_{1}\right]$$

(24) $$\left\{R_{1}\right\}=\{\begin{array}{ll} \beta A_{123}/3{\left[\begin{array}{cccc} 1 & 1 & 1 & 0 \end{array}\right]}^{T} & \text{for flux BCs on face 1-2-3}\\ \beta A_{234}/3{\left[\begin{array}{cccc} 0 & 1 & 1 & 1 \end{array}\right]}^{T} & \text{for flux BCs on face 2-3-4}\\ \beta A_{341}/3{\left[\begin{array}{cccc} 1 & 0 & 1 & 1 \end{array}\right]}^{T} & \text{for flux BCs on face 3-4-1}\\ \beta A_{412}/3{\left[\begin{array}{cccc} 1 & 1 & 0 & 1 \end{array}\right]}^{T} & \text{for flux BCs on face 4-1-2} \end{array}$$

(25) $$\left\{R_{2s}\right\}=\frac{VE_{AC}}{4}\left\{\begin{array}{l} 1\\ 1\\ 1\\ 1 \end{array}\right\}\;\text{if}\;t_{s}\leq t<t_{d}$$

(26) $$\left\{R_{2b}\right\}=\frac{Vb}{4}\left\{\begin{array}{l} 1\\ 1\\ 1\\ 1 \end{array}\right\}\;\text{if}\;t\geq t_{d}$$

In the above equations, (*x_i_, y_i_, z_i_*) are the nodal coordinates of the four-node tetrahedral element under consideration, *V* is the volume of the element, and *A_ijk_* is the area of face *i*–*j*–*k* of the element.

At this point, the matrices and vectors in Eqs. (20)–(26) for all elements need to be expanded to the structure/model size before they can be assembled to obtain the global version of the first-order differential Eq. (11).

By using the time integration method [22], the vector of unknown concentrations {*c*}_*i*+1_ at time *t*_*i*+1_ can be found from {*c*}*_i_* at time *t_i_* as

(27) $$\begin{array}{ll} \left[\frac{1}{\text{Δ}t}\left[K_{1}\right]+\gamma \left[K_{2}\right]\right]{\left\{c\right\}}_{i+1}=\left[\frac{1}{\text{Δ}t}\left[K_{1}\right]-(1-\gamma )\left[K_{2}\right]\right]{\left\{c\right\}}_{i} & \text{if}\;t<t_{s}\\ \begin{array}{l} \left[\frac{1}{\text{Δ}t}\left[K_{1}\right]+\gamma \left[K_{2}\right]\right]{\left\{c\right\}}_{i+1}=\left[\frac{1}{\text{Δ}t}\left[K_{1}\right]-(1-\gamma )\left[K_{2}\right]\right]{\left\{c\right\}}_{i}+\\ \;\;\;\;\;\;\;(1-\gamma ){\left\{R_{1}+R_{2s}\right\}}_{i}+\gamma {\left\{R_{1}+R_{2s}\right\}}_{i+1} \end{array} & \begin{array}{l} \text{if}\;t_{s}\leq t<t_{d} \end{array}\\ \begin{array}{l} \left[\frac{1}{\text{Δ}t}\left[K_{1}\right]+\gamma \left[K_{2}+K_{3}\right]\right]{\left\{c\right\}}_{i+1}=\left[\frac{1}{\text{Δ}t}\left[K_{1}\right]-(1-\gamma )\left[K_{2}+K_{3}\right]\right]{\left\{c\right\}}_{i}+\\ \;\;\;\;\;\;\;(1-\gamma ){\left\{R_{1}+R_{2b}\right\}}_{i}+\gamma {\left\{R_{1}+R_{2b}\right\}}_{i+1} \end{array} & \begin{array}{l} \text{if}\;t\geq t_{d} \end{array} \end{array}\}$$

where $\text{Δ}t=t_{i+1}-t_{i}$ is the time step and in this work, Galerkin’s implicit method (γ=2/3) was chosen as the method is known to be unconditionally stable (no restriction on Δt for obtaining a stable solution, [22]).

The equation system (27) is simply a linear system of algebraic equations of the form

(28) $$[K]{\left\{c\right\}}_{i+1}={\left\{F\right\}}_{i+1}$$

If some nodal concentrations are prescribed, these boundary conditions must be applied to Eq. (28) to obtain a reduced system of linear equations that contains the vector {*c_r_*}_*i*+1_ of only unknown nodal concentrations at time *t*_*i*+1_.

For each step time of the time integration method described in Eq. (27), as mentioned before, an iterative process must be utilized for the quasi-linearization of the Michaelis-Menten model. To be specific, the concentration solution {*c*}*_i_* from the previous step time *t_i_* will be used as an initial guess for *C*_1_ employed in evaluating [*K*_3_] and {*R*_2_} for calculations at step time *t*_*i*+1_ (see Eqs. (6), (23) and (26)). The solution {c*}_*i*+1_ resulting from using these [*K*_3_] and {*R*_2_} is expected to be a better guess for *C*_1_ and this process should be repeated until a chosen convergence criteria is met.

## 4. Validation of the proposed FEA model

The 3-D FEA model developed was validated against some available analytical solutions and published data. The FEA simulations employed the same cAMP signaling data as in [17] for PMVECs, *i.e.*, *E*_*AC*_ = 0.1412 *μ*M/s for synthesis, *D* = 0.3 to 300 *μ*m^2^/s for diffusion, *V*_max_ = 0.295 *μ*M/s, *K*_*M*_ = 2 *μ*M for degradation, and *C*_*o*_ = 0.05 *μ*M for initial conditions. In addition, no cAMP signal is assumed to be transported between the cytoplasm and the nucleus.

**Fig. 2.**
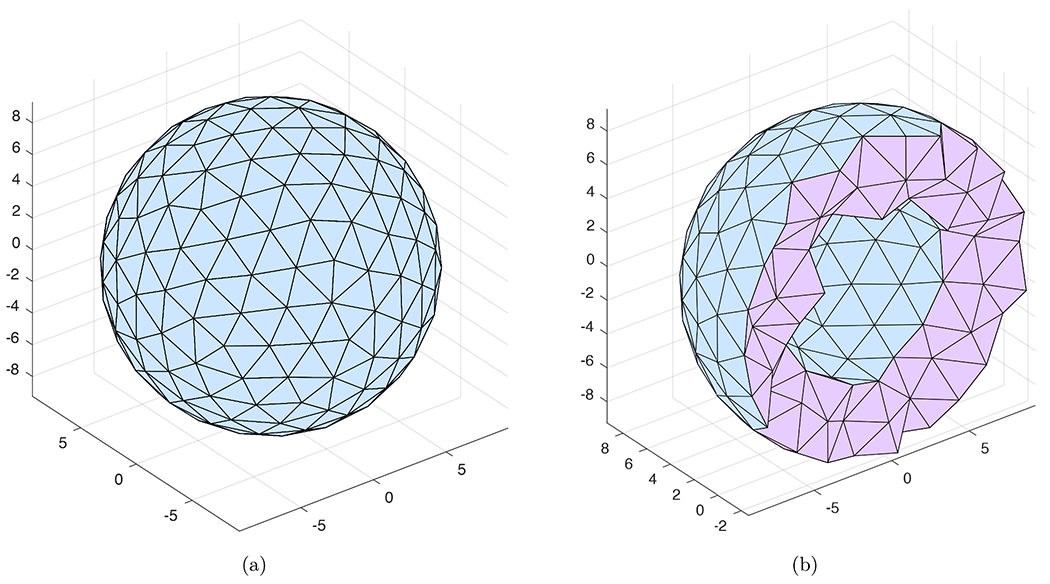
Tetrahedral mesh for a spherical cell model with units in *μ*m: (a) surface mesh; (b) cross section.

The validation was first conducted against some analytical solutions. For this purpose, FEA results were obtained using a spherical cell model. The radii of the cell and the nucleus are *R*_*o*_ = 9.34 *μ*m and *R_i_* = 5.26 *μ*m, respectively. Fig. 2(a) and 2(b) show the tetrahedral meshes with 381 nodes and 1,472 elements for this spherical cell model. These meshes were constructed by Distmesh [23] (a MATLAB program developed by Per-Olof Persson for generating and manipulating unstructured 2-D and 3-D meshes).

**Fig. 3.**
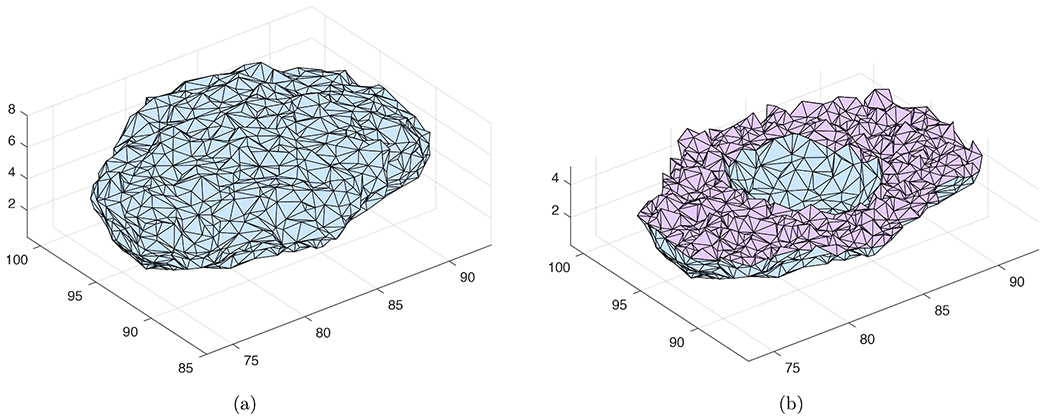
Tetrahedral mesh for a cultured PMVEC model with units in *μ*m: (a) surface mesh; (b) cross section.

The validated 3-D FEA model was then applied to simulate intra cellular cAMP signaling within a cultured PMVEC model. Fig. 3(a) and 3(b) depict the tetrahedral meshes with 4,983 nodes and 29,069 elements for the PMVEC model.

The FEA meshes shown in Fig. 2(a), 2(b), 3(a) and 3(b) are those satisfying the mesh convergence tests presented in section 4.1.2. These meshes also have good quality measured by their aspect ratio and solid angle as depicted in Table 1 [24]. For each quality measure, its best and worst possible values are 1 and 0, respectively. The mesh quality for the spherical cell model is better than the PMVEC model as the geometry of the latter is much more complex.

### 4.1. Convergence tests

#### 4.1.1. Time-step convergence tests

**Fig. 4.**
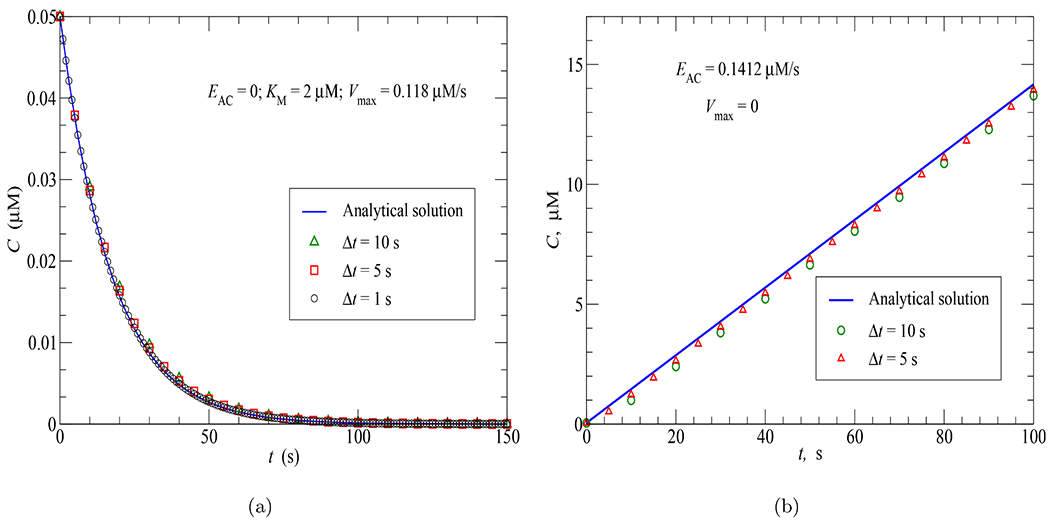
Time-step convergence tests: (a) Spherical cell model; (b) PMVEC model.

As mentioned above, the Galerkin’s implicit method chosen has no restriction on the time step Δ*t* for producing stable solutions. To illustrate this advantage, two time-step convergence tests were performed: the first one for the cAMP concentration response of the spherical cell model to PDE activity uniformly distributed in the cytosolic region (see section 4.2.1 for more details) and the second one for the cAMP concentration response of the PMVEC model to AC activity uniformly distributed in the cytosol (see section 4.2.2). The results in Fig. 4(a) and 4(b) confirm the advantage of Galerkin’s implicit method as the cAMP responses remain stable with different time steps selected, namely, Δ*t* = 10, 5 and 1 sec. For the spherical cell model using mesh 3 (see Table 2 and section 4.1.2), the numerical results are mostly unchanged and accurate even with the use of a very coarse time step of 10 sec. For the PMVEC model with a complex geometry (934 nodes and 5,039 elements), the error of the numerical results is visible at large time steps. However, the accuracy of the FEA result in case Δ*t* = 10 sec is still acceptable. As expected, the results converge to the analytical solution as Δ*t* is reduced.

#### 4.1.2. Mesh convergence tests

A series of meshes with increasing mesh density were employed for the mesh convergence tests. Table 2 shows the information for the two coarsest and two finest meshes in the series for the spherical cell model.

**Fig. 5.**
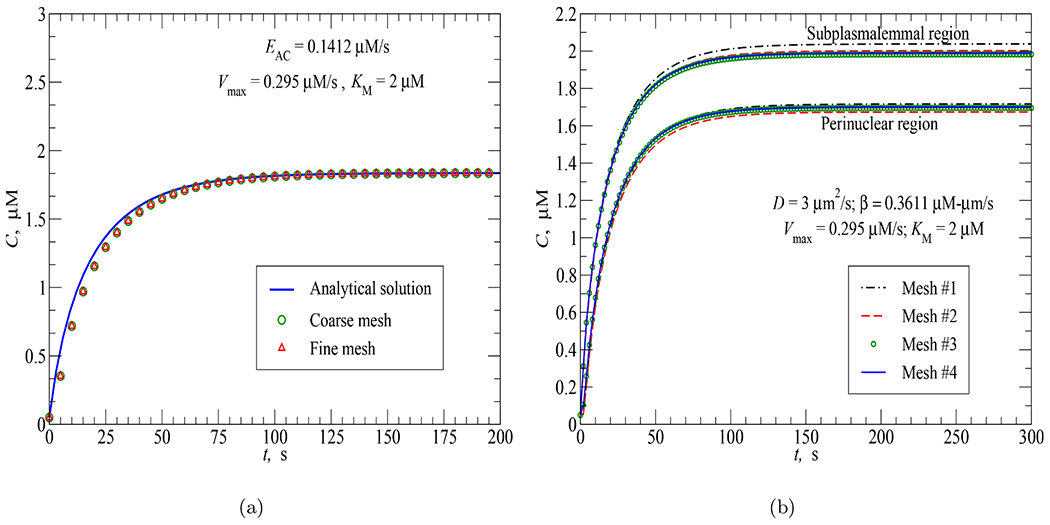
Mesh convergence tests: (a) PMVEC model; (b) Spherical cell model.

For compartmental models (see sections 4.2.1 and 4.2.2) the outputs are almost independent of the mesh density. As a result, the use of mesh 1 (see Table 2) was sufficient for obtaining the accurate FEA results shown in sections 4.2.1 and 4.2.2. The result of the mesh convergence test on the PMVEC model shown in Fig. 5(a) demonstrates this mesh independence behavior of compartmental models. In this test, the coarse mesh has 934 nodes and 5,039 tetrahedral elements while the fine mesh is shown in Fig. 3(a) and 3(b).

For non-compartmental models, such as those presented in section 4.3, the outputs are sensitive to the mesh density. Fig. 5(b) depicts the time courses of cAMP concentration in the spherical cell model at the subplasmalemmal and perinuclear regions in response to AC activity uniformly distributed over the plasma membrane (see section 4.3 for more detailed information). As the mesh density increases, it can be seen that the solutions converge. Since the FEA results obtained from meshes 3 and 4 are almost the same, mesh 3 were selected for all the FEA simulations in sections 4.2.3 and 4.3.

### 4.2. Validation against analytical solutions

There is no general analytical solution to the governing Eq. (1). However, analytical solutions are available for some particular cases involving simple cellular geometries or uniform loading conditions. These particular cases are considered in this section to partially verify the proposed FEA model.

#### 4.2.1. Verification of the implementation of the Michaelis-Menten equation

If there is no concentration flux (*β* = 0) at the subplasmalemmal and perinuclear regions, and cAMP synthesis and/or degradation occur uniformly in the cytosolic region, then there is no diffusion of cAMP which means cAMP concentration at any cellular location is the same at a given time. This is known as a compartmental model for which the resulting concentration *C* is independent of cellular location (*x, y, z*), cellular geometries and the diffusion coefficient *D* which means that *C* is a function of time only. Hence, for $t\geq t_{d}$, the partial differential Eq. (1) becomes the following ordinary differential equation for finding *C*(*t*):

(29) $$\frac{\text{d}C}{\text{d}t}=E_{AC}-\frac{V_{\max}C}{K_{M}+C}$$

The analytical solution for this equation is given by Eq. (33) in the Appendix.

**Fig. 6.**
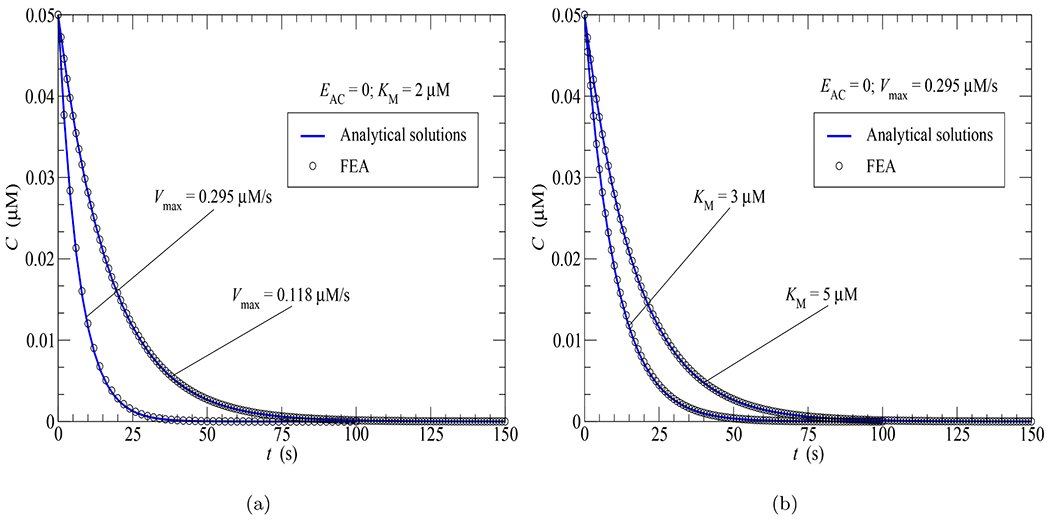
FEA vs analytical solutions for the time course of cAMP concentration in response to only PDE activity uniformly distributed in the cytosol: (a) Effect of *V*_max_; (b) Effect of *K*_*M*_.

In this section, Eq. (29) was used to validate the FEA implementation of the iterative process for the quasi-linearization of the Michaelis-Menten Eq. (2). To this end, a compartmental model for which no AC activity (*E*_*AC*_ = 0) is present and PDE activity is uniformly distributed within the cytosol from *t_d_* = 0 was studied. The concentration responses for four different scenarios of PDE activities are depicted in Fig. 6(a) and 6(b) where the FEA results agree perfectly with the analytical solutions given by Eq. (35). The outputs confirm the compartmental behavior of the FEA model as they are independent of the cellular position, the cellular geometry (PMVEC or spherical cell) and the diffusion coefficient used. As predicted, an increase of *V*_max_ with respect to *K*_*M*_ (Fig. 6(a)) speeds up the cAMP degradation, while an elevation of *K*_*M*_ with respect to *V*_max_ slows down the degradation.

As with the 2-D FEA model [16], the following biological behavior can be observed for compartmental models studied in this section: cAMP concentration responses are the same as long as the ratio of *V*_max_ */K*_*M*_ is the same. This can be confirmed by noticing that the curve for *V*_max_ = 0.118 *μ*M/s and *K_M_* = 2 *μ*M in Fig. 6(a) and the curve for *V*_max_ = 0.295 *μ*M/s and *K*_*M*_ = 5 *μ*M in Fig. 6(b) are identical as they have the same ratio *V*_max_/*K*_M_ = 0.059.

#### 4.2.2. Verification of the implementation of cAMP synthesis

Eq. (29) was also employed to validate the implementation of cAMP synthesis in the proposed FEA model. This was done by using a compartmental scenario where the plasma membrane and the perinuclear region are under flux-free boundary conditions, PDE activity is absent (*V*_max_ = 0) and AC activity is uniformly occurred inside the cytosol from *t*_*s*_ = 0. The analytical solution for the compartmental output for this case is given by Eq. (36)

**Fig. 7.**
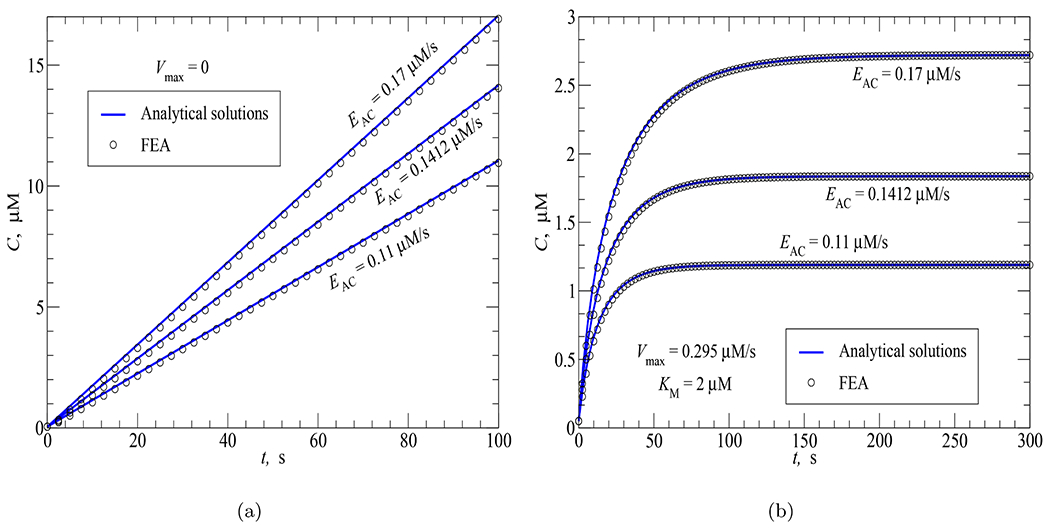
FEA vs analytical solutions for the time course of cAMP concentration in response to (a) AC activity uniformly distributed in the cytosol; (b) AC and PDE activities uniformly distributed in the cytosol.

Three AC activities characterized by three synthesis rates *E_AC_* = 0.11, 0.1412 and 0.17 *μ*M/s were investigated. Very good agreement between the FEA results and the analytical solutions can be seen in Fig. 7(a).

In a next step, a further validation involving a compartmental case where both AC and PDE activities take place at the same time was considered. This was done by adding to the three AC activities a uniform PDE activity (*V*_max_ = 0.295 *μ*M/s, *K*_*M*_ = 2 *μ*M) within the cytosol from *t*_*d*_ = 0. Fig. 7(b) shows an excellent agreement between the FEA results and the analytical counterparts obtained by employing Eq. (33). As expected, a lower amount of cAMP synthesis rate results in a shorter time before the steady state of concentrations being reached.

#### 4.2.3. Verification of the implementation of cAMP diffusion

To validate the proposed FEA model in terms of numerical implementation of cAMP diffusion, two simulations involving different boundary conditions at the subplasmalemmal and perinuclear regions were studied. Both simulations were conducted on the spherical cell geometry in Fig. 2(a) subjected to a diffusion coefficient *D* = 30 *μ*m^2^/s and an absence of AC and PDE activities within the cytosol (*E*_*AC*_ = 0 and *V*_max_ = 0).

**Fig. 8.**
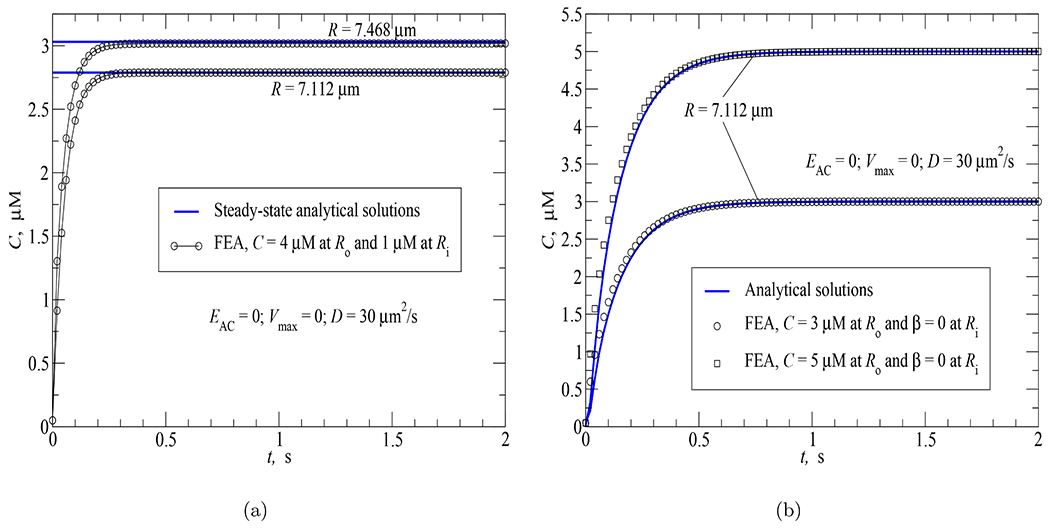
FEA vs analytical solutions for the time courses of cAMP concentration at different cellular locations in response to diffusion coefficient *D* = 30 *μ*m^2^/s and different boundary conditions: (a) *C* = 4 *μ*M at the plasma membrane and *C* = 1 *μ*M at the perinuclear region; (b) *C* = 3 *μ*M or 5 *μ*M at the plasma membrane and *β* = 0 at the perinuclear region.

For the first simulation, cAMP concentrations of 4 *μ*M and 1 *μ*M were prescribed over the plasma membrane and perinuclear region, respectively. The FEA results for the time response of cAMP concentration on two spherical surfaces of radii *R* = 7.468 *μ*m and *R* = 7.112 *μ*m within the cytosol are favorably compared against the steady-state analytical results as shown in Fig. 8(a).

For the second simulation, concentration boundary conditions (*C* = 3 *μ*M or *C* = 5 *μ*M) were applied at the plasma membrane while flux-free boundary conditions were imposed over the perinuclear region. Because of these boundary conditions, the cAMP concentration at any locations in the subplasmalemmal region will remain at the applied value (3 *μ*M or 5 *μ*M) while the concentration at other locations in the cytosolic and perinuclear regions will rise from the initial value of 0.05 *μ*M to the steady-state value of 3 *μ*M or 5 *μ*M. Due to this behavior, it is sufficient to show the FEA result for a representative location within the cytosolic region.

Fig. 8(b) depicts the FEA vs analytical results for the time responses of cAMP concentration on the spherical surface of radius *R* = 7.112 *μ*m within the cytosol under two cases of concentration boundary conditions aforementioned. For both cases, the FEA results agree very well with the analytical solutions available in [25].

### 4.3. Validation against other numerical technique

In this section, two simulations involving non-compartmental models for the spherical cell geometry (Fig. 2(a) and 2(b)) were used to verify the proposed FEA technique as a whole by comparing the FEA results with those obtained from the finite volume technique implemented within the VCell software [15].

In both simulations, AC activity was uniformly distributed at the plasma membrane and PDE activity was uniformly distributed in the cytosol. The first simulation employed a diffusion coefficient of 3 *μ*m^2^/s while that value chosen for the second simulation was 0.3 *μ*m^2^/s. Note that these simulations were previously run using the VCell software and the results were reported in [17].

The uniform AC distribution at the subplasmalemmal region was modeled using a positive flux on the outer surface of the spherical cell geometry. As in [17], *β* was determined under the assumption of the same total AC activity produced either over the plasma membrane or inside the cytosolic region, *i.e.*,

(30) $$E_{AC}V_{c}=\beta S_{p}$$

where *E*_*AC*_ = 0.1412 *μ*M/s, *V*_*c*_ and *S*_*p*_ are the volume of the cytosolic region and surface area of the plasma membrane of the spherical cell geometry shown in Fig. 2(a).

Therefore,

(31) $$\beta =\frac{E_{AC}V_{c}}{S_{p}}=\frac{E_{AC}\left(R_{o}^{3}-R_{i}^{3}\right)}{3R_{o}^{2}}=0.3611\;\mu \text{M⋅}\mu \text{m}/\text{s}$$

**Fig. 9.**
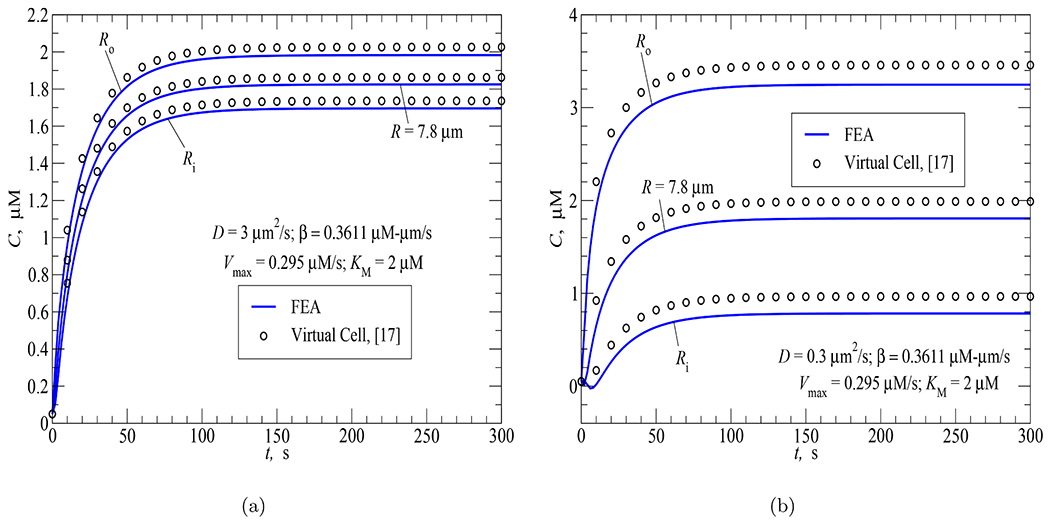
FEA vs finite volume (Virtual Cell) solutions for the time courses of cAMP concentration at the subplasmalemmal, cytosolic and perinuclear regions in response to AC activity (*β* = 0.3611 *μ*M·*μ*m/s) uniformly distributed over the plasma membrane and PDE activity uniformly distributed in the cytosol (a) *D* = 3 *μ*m^2^/s; (b) *D* = 0.3 *μ*m^2^/s.

Fig. 9(a) and 9(b) show a comparison between the FEA and VCell results for the first (*D* = 3 *μ*m^2^/s) and second (*D* = 0.3 *μ*m^2^/s) simulations, respectively. In each figure, the time responses of cAMP concentration on the plasma membrane (*R*_*o*_), on the spherical surface of radius *R* = 7.8*μ*m and at the perinuclear region (*R*_*i*_) are plotted. There is reasonable agreement between the two results (FEA vs VCell), but not perfect. For the second simulation (see Fig. 9(b)), some noticeable discrepancy occurs at the perinuclear region in the early time of the process (*t* < 10 s). However, the FEA curve during this early time period makes sense as the perinuclear region is far from the source of synthesis while *D* is small (cAMP spread is slow): cAMP concentration at the perinuclear region should first drop from the initial value of 0.05 *μ*M (due to PDE activity) before AC activity reaches this region and raises *C* to its steady-state value. The absence of this concentration drop on the VCell curve may be explained by the fact that the VCell simulation in [17] used a larger time step (Δ*t* = 10 s vs Δ*t* = 2 s employed in the FEA simulation) which prevented it to capture the concentration drop within the first 10 seconds of the process.

### 4.4. FEA simulations on the 3-D cultured PMVEC geometry

**Fig. 10.**
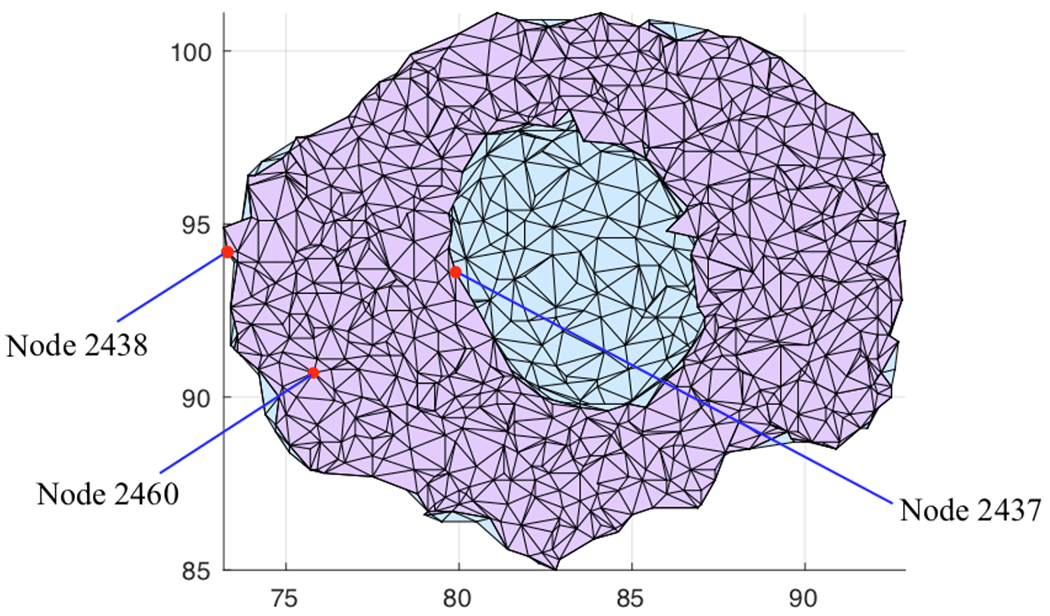
Location of nodes 2438 (on the plasma membrane), 2460 (in the cytosol) and 2437 (at the perinuclear region).

The proposed FEA model previously validated was used in this section to simulate cAMP cellular signaling within the complex 3-D cultured PMVEC geometry shown in Fig. 3(a) and 3(b). Two simulations with and without delayed start of AC and PDE activities were considered herein. The time responses of cAMP concentration at three typical cellular locations were sought. These locations are represented by nodes 2438, 2460 and 2437 at the subplasmalemmal, cytosolic and perinuclear regions, respectively. These nodes have the same *z*-coordinate of 4.6 *μ*m. The location of these nodes in the cross section *z* = 4.6 *μ*m is depicted in Fig. 10.

#### 4.4.1. Uniform AC and PDE activities

In this simulation, AC activity uniformly generated from the plasma membrane was modeled using a positive flux *β* = 2 *μ*M·*μ*m/s. PDE activity (*V*_max_ = 0.295 *μ*M/s, *K*_*M*_ = 2 *μ*M) was uniformly taken place throughout the cytosol. A diffusion coefficient *D* = 10 *μ*m^2^/s was selected. There was no delayed start of AC and PDE activities, *t*_*s*_ = *t_d_* = 0s.

**Fig. 11.**
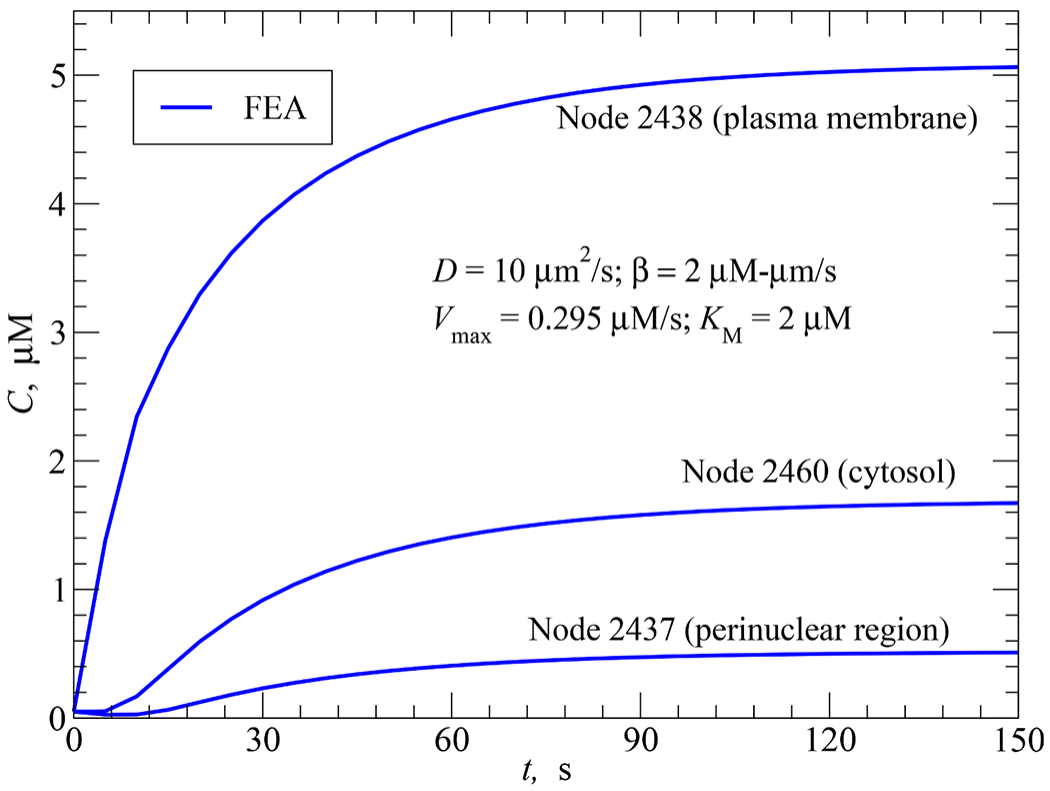
FEA solution for the time responses of cAMP concentration at nodes 2438 (on the plasma membrane), 2460 (in the cytosol) and 2437 (at the perinuclear region) in response to AC activity (*β* = 2 *μ*M·*μ*m/s) uniformly distributed over the plasma membrane at *t_s_* = 0, PDE activity uniformly distributed in the cytosol at *t_d_* = 0, and *D* = 10 *μ*m^2^/s.

Fig. 11 shows the time responses of cAMP concentration at three chosen cellular locations. As expected, all the three curves start from the initial conditions and then approach their respective steady-state values. The closer the node to the source of synthesis, the higher the amount of steady-state concentration. As nodes 2460 and 2437 are at some distances from the plasma membrane where AC activity occurs, their curves exhibit an initial drop of cAMP concentration due to cAMP degradation before they recover and reach steady-state level.

#### 4.4.2. Uniform AC and PDE activities with delayed start

**Fig. 12.**
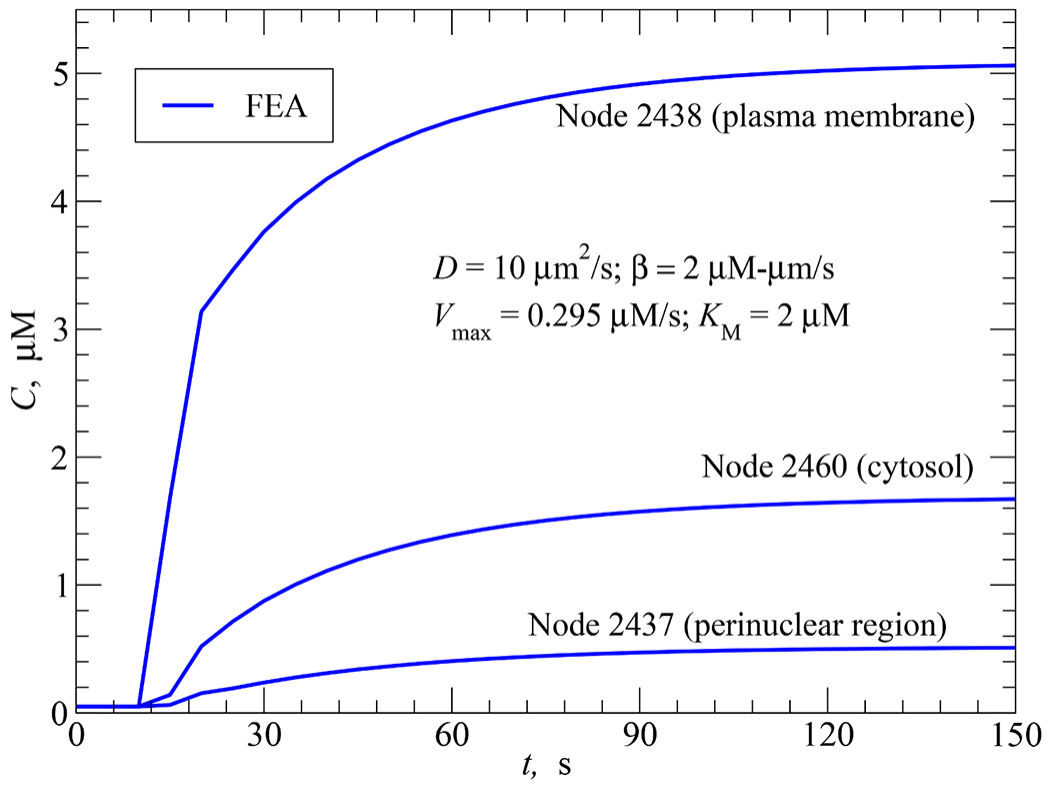
FEA solution for the time responses of cAMP concentration at nodes 2438 (on the plasma membrane), 2460 (in the cytosol) and 2437 (at the perinuclear region) in response to AC activity (*β* = 2 *μ*M·*μ*m/s) uniformly distributed along the plasma membrane at *t_s_* = 10 s, PDE activity uniformly distributed in the cytosol at *t_d_* = 20 s, and *D* = 10 *μ*m^2^/s.

The only difference between this and the previous simulation is the delayed start of cAMP synthesis and degradation: AC and PDE activities were assumed to start at *t*_*s*_ = 10 s and *t_d_* = 20 s, respectively. As a result, the concentrations at the three cellular locations remained at the initial level of 0.05 *μ*M until *t* = 10 s when they started to rise (see Fig. 12). Due to cAMP synthesis without degradation between *t* = 10 and 20 s, there is a linear portion within this time period on each of the three curves depicted in Fig. 12. As soon as PDE activity started at *t* = 20 s, the slopes of the three curves decreased and the concentration started approaching their respective steady-state values.

## 5. Conclusion

A developed 3-D finite element model for cAMP intracellular signaling was presented in this paper. The model produced time course plots of cAMP concentrations at selected nodes using equations governing the synthesis, diffusion, and degradation of the second messenger. The finite element model is capable of simulating multiple AC and PDE activity scenarios. It can also confine cAMP synthesis and degradation to certain areas of the cell such as uniformly distributing both AC and PDE activity within the cytosol and/or bounding AC activity to the plasma membrane with a boundary flux condition. Initial cAMP concentrations, diffusion coefficients, and simulation times are also modifiable variables in the model.

The time course cAMP concentration plots simulated with the spherical cell geometry produced almost identical curves to the analytical literature solutions. Due to the use of two different numerical techniques (FEM vs FVM), there was a minor but uniform discrepancy between the simulated FEA and VCell results when a boundary flux *β* was specified to contain AC activity to the plasma membrane while PDE activity was uniformly distributed within the cytosol. Finally, the validated 3-D FEM model was successfully used to simulate cAMP intracellular signaling within a cultured PMVEC cell subjected to different distributions of AC and PDE activities.

In general, similar to a conclusion in our previous 2-D work [16], 3-D simulations also showed that sustained cAMP gradients can be formed within endothelial cells which is consistent with those observed in rat PMVECs [26]. These data demonstrate that the finite element modeling approach is a viable approach for modeling second messenger signaling systems in four dimensions (*x, y, z, t*). While the simulation data employed in this work were acquired from PMVECs, applications of the proposed FEA model to other cell types are possible.

## Figures and Tables

**Table 1:** Mesh quality measures.

Mesh	Measure	Minimum	Maximum	Mean
Spherical cell	Aspect ratio	0.025	0.997	0.880
	Solid angle	0.023	0.961	0.715
PMVEC	Aspect ratio	0.007	0.996	0.547
	Solid angle	0.003	0.987	0.315

**Table 2:** Four representing tetrahedral meshes employed in the mesh convergence test for the spherical cell model.

Mesh #	Number of nodes	Number of elements
Mesh 1	77	271
Mesh 2	97	270
Mesh 3	381	1472
Mesh 4	456	1830

## Appendix

The solution of the following initial value problem:

(32)
$$\frac{\text{d}C}{\text{d}t}=E_{AC}-\frac{V_{\max}C}{K_{M}+C};\;C(0)=C_{0}$$

where *E*_*AC*_, *V*_max_, *K*_*M*_ and *C*_0_ are constants, is given by

(33)
$$C(t)=\frac{K_{M}}{V_{\max}-E_{AC}}\left[E_{AC}+V_{\max}W\left(-\frac{1}{V_{\max}K_{M}}\exp(U)\right)\right]$$

where *W*() is the Lambert *W* function, and

(34)
$$U=\frac{1}{V_{\max}K_{M}}\left[{\left(E_{AC}-V_{\max}\right)}^{2}\left(\frac{BV_{\max}K_{M}}{A}-t\right)-E_{AC}K_{M}\right]\;A=E_{AC}^{2}-V_{\max}\left(2E_{AC}-V_{\max}\right)\;B=\ln\left\{\left(E_{AC}K_{M}+E_{AC}C_{0}-V_{\max}C_{0}\right)\exp\left[\frac{C_{0}}{K_{M}}\left(1-\frac{E_{AC}}{V_{\max}}\right)\right]\right\}$$

If *E_AC_* = 0, the solution reduces to

(35)
$$C(t)=K_{M}W\left(\frac{C_{0}}{K_{M}}\exp\left[\frac{1}{K_{M}}\left(-V_{\max}t+C_{0}\right)\right]\right)$$

If *V*_max_ = 0, the solution is simply given by

(36)
$$C(t)=E_{AC}t+C_{0}$$

## References

[1] Zaccolo M, Zerio A, Lobo MJ, Subcellular organization of the cAMP signaling pathway, Pharmacol. Rev 73 (2021) 278–309. 33334857 10.1124/pharmrev.120.000086 PMC7770493

[2] Yarwood SJ, Special issue on “new advances in cyclic AMP signalling”– an editorial overview, Cells 9 (2020) 2274. 10.3390/cells9102274 PMC7599692 33053803

[3] Serezani CH, Ballinger MN, Aronoff DM, Peters-Golden M, Cyclic AMP: master regulator of innate immune cell function, Am. J. Respir. Cell Mol. Biol 39 (2008) 127–132. 18323530 10.1165/rcmb.2008-0091TR PMC2720142

[4] Penn RB, Embracing emerging paradigms of g protein-coupled receptor agonism and signaling to address airway smooth muscle pathobiology in asthma, Naunyn. Schmiedebergs Arch. Pharmacol 378 (2008) 149–169. 18278482 10.1007/s00210-008-0263-1

[5] Bobin P, Belacel-Ouari M, Bedioune I, Zhang L, Leroy J, Leblais V, Fischmeister R, Vandecasteele G, Cyclic nucleotide phosphodiesterases in heart and vessels: a therapeutic perspective, Arch. Cardiovasc. Dis 109 (2016) 431–443. 27184830 10.1016/j.acvd.2016.02.004

[6] Yang H, Yang L, Targeting cAMP/PKA pathway for glycemic control and type 2 diabetes therapy, J. Mol. Endocrinol 57 (2016) R93–R108. 27194812 10.1530/JME-15-0316

[7] Knott EP, Assi M, Rao SNR, Ghosh M, Pearse DD, Phosphodiesterase inhibitors as a therapeutic approach to neuroprotection and repair, Int. J. Mol. Sci 18 (2017) 696. 10.3390/ijms18040696 PMC5412282 28338622

[8] Rich TC, Webb KJ, Leavesley SJ, Perspectives on: cyclic nucleotide microdomains and signaling specificity: can we decipher the information content contained within cyclic nucleotide signals? J. Gen. Physiol 143 (2014) 17–27. 24378904 10.1085/jgp.201311095 PMC3874573

[9] Rich TC, Karpen JW, Cyclic AMP sensors in living cells: what signals can they actually measure? Ann. Biomed. Eng 30 (2002) 1088–1099. 12449769 10.1114/1.1511242

[10] Rich TC, Xin W, Mehats C, Hassell KA, Piggott LA, Le X, Karpen JW, Conti M, Cellular mechanisms underlying prostaglandin-induced transient cAMP signals near the plasma membrane of HEK-293 cells, Am. J. Physiol. - Cell Physiol 292 (2007) C319–C331. 16899551 10.1152/ajpcell.00121.2006 PMC4712347

[11] Rich TC, Fagan KA, Nakata H, Schaack J, Cooper DM, Karpen JW, Cyclic nucleotide-gated channels colocalize with adenylyl cyclase in regions of restricted cAMP diffusion, J. Gen. Physiol 116 (2000) 147–161. 10919863 10.1085/jgp.116.2.147 PMC2229499

[12] Rich TC, Fagan KA, Tse TE, Schaack J, Cooper DMF, Karpen JW, A uniform extracellular stimulus triggers distinct cAMP signals in different compartments of a simple cell, Proc. Natl. Acad. Sci 98 (23) (2001) 13049–13054. 11606735 10.1073/pnas.221381398 PMC60822

[13] Oliveira RF, Terrin A, Di Benedetto G, Cannon RC, Koh W, Kim M, Zaccolo M, Blackwell KT, The role of type 4 phosphodiesterases in generating microdomains of cAMP: large scale stochastic simulations, PLoS One 5 (7) (2010) e11725. 20661441 10.1371/journal.pone.0011725 PMC2908681

[14] Saucerman JJ, Greenwald EC, Polanowska-Grabowska R, Mechanisms of cyclic AMP compartmentation revealed by computational models, J. Gen. Physiol 143 (1) (2014) 39–48. 24378906 10.1085/jgp.201311044 PMC3874575

[15] Loew LM, The virtual cell project, Novartis Found. Symp 247 (2002) 151–160. 12539954

[16] Stone N, Shettlesworth S, Rich TC, Leavesley SJ, Phan AV, A two-dimensional finite element model of cyclic adenosine monophosphate (cAMP) intracellular signaling, SN Appl. Sci 1 (2019) 1713. 33615142 10.1007/s42452-019-1757-9 PMC7891547

[17] Feinstein WP, Zhu B, Leavesley SJ, Sayner SL, Rich TC, Assessment of cellular mechanisms contributing to cAMP compartmentalization in pulmonary microvascular endothelial cells, Am. J. Physiol. – Cell Physiol 302 (6) (2012) C839–C852. 22116306 10.1152/ajpcell.00361.2011 PMC3311237

[18] Shetdesworth S, Webb KJ, Phan AV, Rich TC, A 3-d finite element model of the synthesis, degradation, and spatial spread of cAMP. birmingham. 2016 ASME Early Career Technical Journal, 2016.

[19] Stryer L, Biochemistry, W.H. Freeman and Company, New York, 1995.

[20] Rich TC, Fagan KA, Tse TE, Schaack J, Cooper DMF, Karpen JW, A uniform extracellular stimulus triggers distinct cAMP signals in different compartments of a simple cell, Proc. Natl. Acad. Sci 98 (23) (2001) 13049–13054. 11606735 10.1073/pnas.221381398 PMC60822

[21] Bhatti MA, Fundamental Finite Element Analysis and Applications: With Mathematica and Matlab Computations, Wiley, 2005.

[22] Bathe KJ, Finite Element Procedures, Prentice-Hall, Englewood Cliffs, NJ, 1996.

[23] Persson PO, Strang G, A simple mesh generator in MATLAB, SIAM Rev. 46 (2) (2004) 329–345.

[24] Field D, Qualitative measures for initial meshes, Int. J. Numer. Methods Eng 47 (2000) 887–906.

[25] Lü Y, Bülow M, Analysis of diffusion in hollow geometries, Adsorption 6 (2) (2000) 125–136.

[26] Annamdevula NS, Sweat R, Griswold JR, Trinh K, Hoffman C, West S, Deal J, Britain AL, Jalink K, Rich TC, Leavesley SJ, Spectral imaging of FRET-based sensors reveals sustained cAMP gradients in three spatial dimensions, Cytometry A 93 (10) (2018) 1029–1038. 30176184 10.1002/cyto.a.23572 PMC6512796

